# Acute Pancreatitis and Type 2 Diabetes Mellitus: The Chicken–Egg Paradox—A Seven-Year Experience of a Large Tertiary Center

**DOI:** 10.3390/jcm13051213

**Published:** 2024-02-21

**Authors:** Mihai Radu Pahomeanu, Damiana Ojog, Diana Teodora Nițu, Irina Ștefania Diaconu, Hosein Nayyerani, Lucian Negreanu

**Affiliations:** 1 Faculty of Medicine, Carol Davila University of Medicine and Pharmacy, 050474 Bucharest, Romania; 2 Bucharest Acute Pancreatitis Index (BUC-API) Study Group, 077135 Mogoșoaia, Romania; 3 Internal Medicine and Gastroenterology Department, University Emergency Hospital of Bucharest, 050098 Bucharest, Romania

**Keywords:** type 2 diabetes mellitus, acute pancreatitis, severity, ICU

## Abstract

(1) **Background:** Preexisting type 2 diabetes mellitus (T2DM) has been shown in some studies as a risk factor and a severity factor for acute pancreatitis (AP). In this study, we aimed to demonstrate the link between T2DM and AP using data from a large retrospective epidemiological registry in a tertiary center. (2) **Methods:** We conducted a retrospective, large-cohort study of 1855 cases of AP and recurrent AP drawn from the seven-year consecutive hospitalization electronic health records of the largest acute-care tertiary teaching center in Romania. (3) **Results:** We observed a significant association between T2DM and a more severe course of the disease, and between T2DM and admission to the intensive care unit (ICU) due to AP, in our cohort using a chi-square test. However, we did not see a meaningful difference in comparing LoS-ICU between T2DM-AP and OAP (other known cause of AP). AP patients with T2DM had a greater probability of a severe course of the disease and were more likely to be admitted to the ICU than to the OAP. (4) **Conclusions:** The association between T2DM and AP remains a topic very representative of the “chicken–egg paradox”. We need further research on DM-related AP and their bidirectional association as our study is limited by its retrospective design.

## 1. Introduction

In the 1st century CE, when Plutarch introduced the chicken–egg paradox in his essay “The Symposiacs” published in the essay collection Moralia, he was certainly not thinking about which comes first: acute pancreatitis (AP) or type 2 diabetes mellitus (T2DM).

These two diseases are very common—DM had a global estimated prevalence of 538 million people in 2021 [1] out of which 96% represented T2DM, and AP had a global estimated incidence ranging between 2.71 cases-year and 134.9 cases-year per 100,000 people in 2021 [2]. We note a rise of incidence and prevalence in both conditions.

In several observational studies, preexisting T2DM has been linked to an increased risk of AP [3,4], but the association of T2DM with severity of AP has not been as well studied. Moreover, basic animal research has shown that induced diabetes in mice might aggravate AP in terms of enhanced pancreatic inflammatory response, increased pancreatic apoptosis, edema formation, and pulmonary injury [5], possibly through overactivation of the nucleotide-binding domain, leucine-rich-containing family, pyrin domain-containing 3 (NLRP3) pathway [6]. A recent meta-analysis of nine studies showed a link between preexisting T2DM and increased AP severity [7]; unfortunately, only one of the studies [8] used the Revised Atlanta Classification for severity, which, in our opinion, is a significant limitation of the well-designed study due to the heterogeneity of its definition of severity.

There is a current debate regarding the relationship between AP and T2DM. On the one hand, evidence has been presented that AP is a predisposing state for T2DM [9,10]; and on the other hand, some studies have found that diabetes could cause AP [11,12]. 

In this paper, we present epidemiological data on ICU admission and AP severity (as described in the Revised Atlanta Classification [13]) related to T2DM.

Other often-seen risk factors of AP are gallstones, alcohol, hypertriglyceridemia, and drugs [14].

## 2. Materials and Methods

### 2.1. Data Filtering

This study is a retrospective, large cohort study of patients from the Bucharest Acute Pancreatitis Index (BUC-API) registry, which is a registry of 2039 cases of AP, recurrent AP (RAP), and acute-on-chronic pancreatitis (AoCP) from the electronic health records of the University Emergency Hospital of Bucharest (Spitalul Universitar de Urgență București) (SUUB). The BUC-API registry was approved by the SUUB’s Institutional Review Board and informed consent was obtained from all the patients before hospitalization. This study was conducted in accordance with the ethical guidelines of the 1975 Declaration of Helsinki and the Strengthening the Reporting of Observational studies in Epidemiology (STROBE) guidelines.

SUUB is one of the largest acute-care teaching tertiary hospitals in Romania, with 1099 beds and both gastroenterological and abdominal surgery departments.

The cases in the registry were drawn from 35 of the 42 counties in Romania, but 87.3% of the cases were drawn from southern Romania.

For this study, each newly admitted patient was considered a separate case. The cases were selected from the BUC-API registry, all bearing the following International Classification of Disease, 10th edition (ICD-10) codes: K85, B26.3, and B25.2. All of them represented consecutive discharges from 1 June 2015 to 1 April 2022.

A total of 2039 consecutive cases were considered for inclusion in the BUC-API registry. All of them were screened by medical trained staff in order to fulfill positive criteria of diagnosis as stated by Revised Atlanta Criteria [13]. Of these cases, 184 (9.0%) were AoCP cases but were not tagged as miscoded because there is no ICD-10 code for this condition. We considered AoCP to be any case that had positive diagnosis criteria for AP [13] and calcification within pancreas and/or dilation or strictures of Wirsung or Santorini ducts proven by imagistic investigation. The final number of consecutive cases of AP in the registry was 1855 (91.0%).

From the 1855 cases of AP from the BUC-API registry, data were missing regarding tobacco smoking (*n* = 1406, 75.8%), morphology (*n* = 562, 30.3%), and rurality (*n* = 16, 0.9%). Morphology and severity were classified according to the Revised Atlanta Classification [13]. All cases that had missing data were excluded from the statistical analysis regarding that particular topic.

For ease of statistical analysis, we considered only the six most frequently encountered causes of AP, as detailed in Table 1. Whenever there was a mix of etiologies, we reported the case as that of the single most pertinent etiology based on the authors’ consensus. We considered T2DM the cause of AP if there was any biochemical sign of decompensated T2DM or of T2DM with poor therapeutic control and no other obvious known cause of AP. We used the criteria that the American Diabetologist Association set in 2011 for diagnosing new-onset T2DM [15]: HbA1C > 6.5% and/or any random plasma glucose during hospitalization > 200 mg/dL. Cases that had type 1 diabetes mellitus previously diagnosed were excluded from the study.

Cases of idiopathic AP were excluded from the comparative analysis.

AP was considered to have recurred if there was no sign of chronic pancreatitis but the patient had been hospitalized in our hospital for AP in the timespan of the BUC-API registry or had previous episodes of AP, regardless of the former cause, as mentioned explicitly in the EHRs. 

The data regarding the hospitalization costs are reported in Romanian leu (RON).

### 2.2. Statistical Analysis and Software Deployment

The data were organized for this study using Microsoft Office Excel 2019 ©, now known as Microsoft 365 Excel ©, and Google Docs ©. The general characteristics of these data are presented in Table 1 and were analyzed via frequency tests. Moreover, to examine the correlation between two categorical variables, the Pearson chi-square and phi and Cramer’s V were applied. Meanwhile, to assess the correlation between a continuous and another categorical variable, the Mann–Whitney U-test was used. All of the statistical analyses were conducted using IBM SPSS Statistics version 29.0.0.0 ©. Results with a *p* value of <0.05 were considered statistically significant. *p* values were reported up to the third decimal place only when they were close to 0.05.

For reference management, Zotero 6 for Windows and Zotero Connector for Google Chrome were used.

## 3. Results

### 3.1. Population Characteristics

Male patients dominated our study population (*n* = 954, 60.7%).

The median age of all the subjects was 57 years, and their median length of stay in the hospital (LoS) was 7 days (interquartile range (IQR) = 6.0), with a median daily cost of hospitalization (DCH) of RON 920.9 (IQR = 432.5). Most (82.8%) of our cases were first attacks of AP. The etiologies are presented in Table 1. 

The AP patients with T2DM (T2DM-AP patients) accounted for only 3.0% (*n* = 55) of our population. Most (51.4%) of our cases had a mild course of AP, and many of our cases (38.5%) had interstitial AP, but they were healed at discharge (83.0%). The rate of ICU admission was 9.6%.

Regarding our subjects’ environmental data, most (71.8%) of them were from urban settings. We had few data on tobacco smoking, as 75.8% of the cases had no such data. Extensive details of the subjects’ characteristics are in Table 1.

### 3.2. Severity

**Figure 1:**
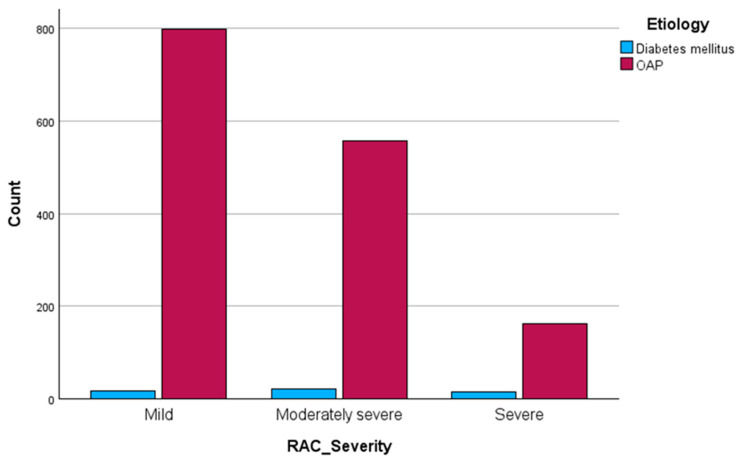
Comparison of the cases related to severity of AP.

The chi-square test revealed a significant association between etiology and severity X2 (df = 2) = 20.9, *p* < 0.01. To check the strength of the association, we calculated Cramer’s V, which was +0.12, suggesting a small strength association between the two variables. Considering that we had a three-level classification of severity, we conducted post hoc analyses using the adjusted standardized residuals (ASRs). We discovered that the ASR with respect to T2DM for the severe AP was +4.2, and for mild AP, −3.2, showing an important difference from the expected frequencies (see Figure 1, Table 2).

### 3.3. ICU Admission

**Figure 2:**
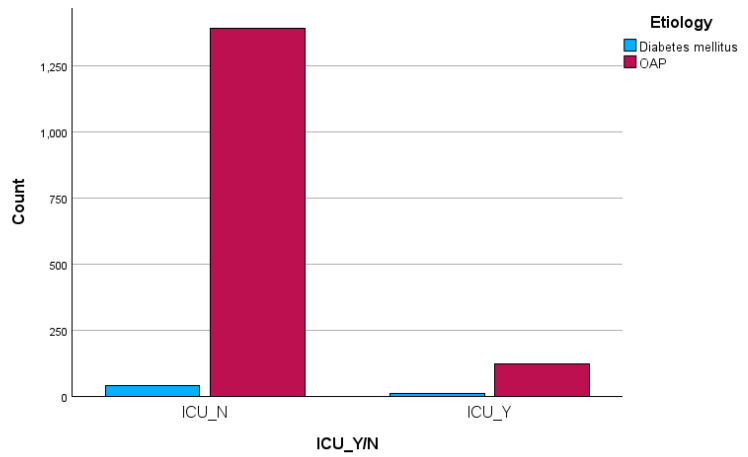
Comparison of the cases regarding ICU admission.

A chi-square test was performed to assess the association between etiology and ICU admission. An important association was seen (X2 (df = 1) = 12.7, *p* < 0.01). To find the strength of the association, we ran Cramer’s V with a value of +0.09, implying a small strength association between the two variables. To further investigate the nature of this association, we examined their post hoc ASR. For ICU admission, we found an ASR of +3.6 for the T2DM-AP cases, which significantly deviates from the expected frequencies. However, the Mann–Whitney U-test did not reveal meaningful disparities concerning LoS-ICU by etiology (U = 655.0, Z = −0.6, *p* = 0.52) (see Figure 2, Table 2).

### 3.4. Secondary Aims

No meaningful variance was found when the chi-square test was run for the following variables: ward of care (X2 (1) = 0.2, *p* = 0.68) with an almost 1:1 ratio about the type of ward that treated the cases. Regarding gender (X2 (1) = 2.3, *p* = 0.13), outcome (X2 (4) = 3.4, *p* = 0.50), recurrence (X2 (1) = 0.1, *p* = 0.97), morphology (X2 (6) = 4.3, *p* = 0.64), tobacco smoking (X2 (2) = 3.4, *p* = 0.18), and rurality (X2 (2) = 2.6, *p* = 0.28), no significant difference betwixt the two analyzed groups was found (see Table 2).

From the Mann–Whitney U-test, we did not observe any substantive disparities between the two etiology groups concerning the following outcomes: age (U = 39,026.5, Z = −0.8, *p* = 0.42); LoS (U = 40,891.5, Z = −0.2, *p* = 0.80); and DCH (U = 25,365.0, Z = 0.0, *p* = 0.98) (see Table 2).

## 4. Discussion

In our registry-based retrospective study that collected data from 1855 consecutive cases, we found an association between T2DM and a more severe course of AP as well as ICU admission.

However, when T2DM-AP and OAP were compared, we did not observe a statistically significant difference in relation to LoS-ICU or mortality.

Paragomi et al. found the same association between DM and severe AP in a prospective international study [11], as did Nawaz et al. in a retrospective study on 7399 cases [16]. A meta-analysis from 2018 [7] and two retrospective studies [8,17] showed an association between DM and a more severe course of disease in AP.

We found that cases of T2DM-AP had higher rates of ICU admission. This result contradicts that of Paragomi et al. [11], who used a large international registry, APPRENTICE, that preexisting DM had no significant impact on the need for ICU admission. The results of other relatively recent studies [8,18,19], including one meta-analysis [7], were similar to ours. The higher rate of ICU admission might be explained by the frailty of DM patients, as many of them suffer from systemic impact complications of DM such as nephropathy, neuropathy, macroangiopathy, and vulnerability to infections.

These conflicting results might be explained by the heterogeneity of the definitions of DM-AP in the studies and of their designs. Particular to Paragomi et al.’s study that we previously mentioned, we consider that although they had run a prospective study, their definition criteria for DM might be flawed. Our fellow researchers took into consideration as T2DM any patient that had “treatment with antidiabetic medication(s)” [11]. It is known that several drugs regarded as antidiabetics have several other indications. In this regard we will mention the following drugs: SGLT-2 inhibitors, used also in both chronic kidney disease with or without DM, and chronic heart failure, both with or without DM and GLP-1 agonists, used also in the treatment of obesity with or without DM. As such, it is highly likely that a proportion of cases taken into consideration by Paragomi et al. could have been mislabeled as DM.

In addition, no meaningful associations were found related to gender in our database, although previous studies showed a higher prevalence of pancreatic diseases in men with DM than in women with DM [20,21]. However, data regarding this topic were scarce. Thus, gender-related data in DM-AP must be expanded in the literature.

Similarly, no association between T2DM-AP and the outcome at discharge was found, although the rate of mortality in the T2DM-AP cases was almost two times higher than that in the OAP cases (9.1% vs. 4.3%, respectively). However, there are controversies related to mortality in DM-AP [22], as Nogaard et al. [23] found that DM is linked to higher long-term mortality, but other studies [16,18] found no relationship between the rate of in-hospital mortality and DM-AP, although DM has been found to be related to a more severe course of AP.

AP recurrence was not found to be linked to T2DM in our cohort, similar to the result of another study [24]. However, we believe that this result should be validated through future prospective studies as our study was a retrospective one with limitations due to its design.

There were no significant differences regarding local complications between T2DM-AP and OAP in this study. Several other studies [7,25], including one meta-analysis, showed an association between DM and local complications in AP, even if the studies did not stratify the results by the type of local complication, as we did in this study. A retrospective small study of 53 cases from 2020 [26] showed an association between DM-AP and WON. However, in all these studies, only the presence of DM in the patient’s medical history was considered in establishing the association.

Currently, tobacco smoking is considered an independent risk factor of AP and RAP [27,28,29], but we found no link between tobacco smoking and T2DM-AP in our population. This could have been because 81.9% of the T2DM-AP cases in our registry had no data regarding smoking. 

We also found no association between T2DM-AP and rurality. Most of our subjects were from urban environments, but more significantly in the T2DM-AP cases (80.0%) than in the OAP cases (70.5%). In countries with a well-developed healthcare system, such as Australia [30], an almost 1:1 ratio of rurality seems to exist; but in medium-income countries, such as China [31,32], rural cases seem prevalent. In our opinion, this rural/urban divide exists in all countries, but Romania might have a high percentage of urban patients because its healthcare system in rural areas is poorly developed.

The median age in our T2DM-AP cases was only slightly higher than in our OAP cases (58 years vs. 56 years). Some studies [3,33] have found that a younger age was more associated with an increased risk of developing AP, but other studies have found the exact opposite, especially regarding male patients [34].

The LoS did not differ significantly by etiology in our study—we found only a slightly shorter median LoS in the T2DM-AP cases than in the OAP cases (6 days vs. 7 days, respectively). A Taiwanese study in 2012 [18] found statistically significant disparities in this regard in a far larger population. Identical median LoS values between DM-AP and OAP cases were also found in a Spanish study [35]. In contrast, a 2018 meta-analysis [7] and a 2015 USA study [16] found a longer LoS in DM-AP cases.

Regarding the median DCH, no significant difference was seen in our cohort, only a slightly smaller median DCH in the T2DM-AP cases than in the OAP cases (RON 874.3 vs. RON 930.9, respectively). We could not find any study on the DCH for DM-AP, but we found a paper on the total hospitalization costs in DM-AP cases—Weissman et al. [20] discovered that DM-comorbid AP cases had a higher average hospitalization cost than non-DM-comorbid AP cases (USD 9934 vs. USD 8486, respectively).

Our T2DM-AP cases had an almost 1:1 ratio of admissions between the gastroenterological department and surgical departments for cases comparable with OAP. However, we did not find recent data (after 2013) regarding the distribution of such cases to different types of wards with which to compare our data. Our admission distribution is specific to Romania’s medical system, in which gastroenterologists and surgeons dispute where to admit AP cases. Recently, though, most AP cases are being admitted to gastroenterological wards.

The strengths of this study are the large population of our registry (*n* = 2039 cases of AP, RAP, and AoCP) and the low bias in our selection of cases, as they were all consecutive hospitalizations within a well-defined timeframe. Possible limitations of this study concern its retrospective design (i.e., missing data on tobacco smoking, morphology, and rurality), nonstratification of the cases according to the type of DM, and our nonaccounting for all DM-comorbid cases, but only for those that did not have any other more probable cause.

The bidirectional relation between DM and AP is a topic highly debated by the medical literature as some treaties in gastroenterology regard it as a possible etiology (see Sleisienger and Fordtran’s Gastrointestinal and Liver Disease 10th edition), whilst other fellow researchers see it as a complication of pancreatic diseases, mostly chronic pancreatitis. We consider that further basic studies, randomized clinical trials, and meta-analysis on this topic can clarify better this “chicken–egg paradox”.

## 5. Conclusions

In our registry-based retrospective study, we found a statistically significant association of T2DM with a more severe course of AP and a higher ICU admission rate of the AP patients, but not with their longer ICU stay. As stated in the introduction and discussion, the association between T2DM and AP remains a topic very representative of the “chicken–egg paradox”. We need further research on DM-related AP and their bidirectional association as our study is limited by its retrospective design.

## Figures and Tables

**Table 1:** Population characteristics.

Acute pancreatitis (AP) and recurrent AP (RAP) cases	1855
Recurrence	
AP (first known attack)	1536 (82.8%)
RAP	319 (17.2%)
Age (years)	
Median	57 (IQR = 26.0)
Mean	56.9 (SD = 17.1)
Days of hospitalization	
Median	7.0 (IQR = 6.0)
Mean	8.8 (SD = 7.8)
Daily cost of hospitalization (RON)	
Median	920.9 (IQR = 432.5)
Mean	2153.1 (SD = 17,129.9)
Etiology	
Type 2 diabetes mellitus (T2DM)	55 (3.0%)
Idiopathic	283 (15.2%)
All other known causes, such as:	1517 (81.8%)
- Biliary (gallstones)	732 (39.5%)
- Alcohol	628 (33.9%)
- Hypertriglyceridemia	58 (3.1%)
- Specific drugs	30 (1.6%)
- Trauma	19 (1.0%)
- Other known causes	50 (2.7%)
Sex	
Male	1098 (59.2%)
Female	757 (40.8%)
Severity	
Mild	954 (51.4%)
Moderate	677 (36.5%)
Severe	224 (12.1%)
Morphology	
Interstitial	715 (38.5%)
Normal pancreas	274 (14.8%)
APFC	136 (7.3%)
ANC	87 (4.7%)
Pseudocyst	76 (4.1%)
WON	5 (0.3%)
No data	562 (30.3%)
Outcome	
Healed/ameliorated	1540 (83.0%)
Discharged at will	116 (6.3%)
Deceased	108 (5.8%)
Transferred	79 (4.3%)
Stationary	12 (0.6%)
ICU	
No	1676 (90.4%)
Yes	179 (9.6%)
Tobacco smoking	
Active	324 (17.5%)
Former (>4 weeks)	90 (4.9%)
Never	35 (1.9%)
No data	1406 (75.8%)
Ward of origin	
Gastroenterology	941 (50.7%)
Surgery	914 (49.3%)
Place of origin	
Urban	1332 (71.8%)
Rural	507 (27.3%)
No data	16 (0.9%)

**Table 2:** Clinical and demographic characteristics of the T2DM-AP versus OAP cases.

	T2DM-AP (*n* = 55)	OAP (*n* = 1517)	*p* Value
Severity			
Mild	17 (30.9%)	798 (52.6%)	*p* < 0.01
Moderately severe	22 (40.0%)	557 (36.7%)	
Severe	16 (29.1%)	162 (10.7%)	
ICU			
No	43 (78.2%)	1394 (91.9%)	*p* < 0.01
Yes	12 (21.8%)	123 (8.1%)	
Ward of care			
Gastroenterology	29 (52.7%)	757 (49.9%)	*p* = 0.68
Surgery	26 (47.3%)	760 (50.1%)	
Gender			
Male	28 (50.9%)	926 (61.0%)	*p* = 0.13
Female	27 (49.1%)	591 (39.0%)	
Outcome			
Healed/ameliorated	45 (81.8%)	1273 (83.9%)	*p* = 0.50
Stationary	0 (0.0%)	11 (0.7%)	
Transferred	2 (3.6%)	71 (4.7%)	
Discharged at will	3 (5.5%)	97 (6.4%)	
Deceased	5 (9.1%)	65 (4.3%)	
Recurrence			
First attack	45 (81.8%)	1244 (82.0%)	*p* = 0.97
Recurrence	10 (18.2%)	273 (18.0%)	
Morphology			
Interstitial	21 (38.2%)	602 (39.7%)	*p* = 0.64
APFC	4 (7.3%)	115 (7.6%)	
Pseudocyst	0 (0.0%)	60 (4.0%)	
ANC	4 (7.3%)	57 (3.8%)	
WON	0 (0.0%)	4 (0.3%)	
Normal pancreas	8 (14.5%)	234 (15.4%)	
No data	18 (32.7%)	445 (29.3%)	
Tobacco smoking			
Active	7 (12.7%)	285 (18.8%)	*p* = 0.18
Former	1 (1.8%)	80 (5.3%)	
Never	2 (3.6%)	24 (1.6%)	
No data	45 (81.9%)	1128 (74.3%)	
Rurality			
Urban	44 (80.0%)	1070 (70.5%)	*p* = 0.28
Rural	11 (20.0%)	432 (28.5%)	
No data	0 (0.0%)	15 (1.0%)	
Age (years)			
Mean	58.6 (SD = 13.7)	56.7 (SD = 17.0)	*p* = 0.42
Median	58 (IQR = 20)	56 (IQR = 25)	
Length of hospital stay (days)			
Mean	9.2 (SD = 7.6)	8.7 (SD = 7.3)	*p* = 0.80
Median	6 (IQR = 7)	7 (IQR = 5.5)	
Length of ICU stay (days)			
Mean	4.2 (SD = 4.3)	5.3 (SD = 5.5)	*p* = 0.52
Median	3.5 (IQR = 2.7)	3 (IQR = 5)	
Daily hospitalization cost (RON)			
Mean	1164.9 (SD = 1265.3)	2173.9 (SD = 17,640.5)	*p* = 0.98
Median	874.3 (IQR = 518.5)	930.9 (IQR = 420.5)	

## Data Availability

Data available upon reasonable request from the corresponding author.

## Abbreviations

CE	Christ (common) era
AP	acute pancreatitis
DM	diabetes mellitus
T2DM	type 2 diabetes mellitus
NLRP3	Nucleotide-binding domain, leucine-rich-containing family, pyrin domain-containing 3
RCT	randomized clinical trial
ICU	intensive care unit
BUC-API	Bucharest Acute Pancreatitis Index
ICD-10	International Classification of Diseases, 10th edition
EHR	electronic health record
RAP	recurrent acute pancreatitis
RON	International Organization for Standardization code of Romanian leu (currency of Romania)
DCH	daily cost of hospitalization
LoS	length of stay
APFC	acute peripancreatic fluid collection
ANC	acute necrotic collection
WON	walled-off necrosis
ASR	adjusted standardized residual
OAP	other known cause of acute pancreatitis
AoCP	acute-on-chronic pancreatitis
